# Inflammatory diet, gut microbiota and sensorineural hearing loss: a cross-sectional and Mendelian randomization study

**DOI:** 10.3389/fnut.2024.1458484

**Published:** 2024-08-16

**Authors:** Yixuan Wang, Jiayi Nie, Kaige Yan, Jing Wang, Xin Wang, Yuxiang Zhao

**Affiliations:** ^1^Department of Otolaryngology Head and Neck Surgery, Shaanxi Provincial People’s Hospital, Xi’an, China; ^2^Xi’an University of Technology, Xi’an, China; ^3^Northwest A&F University, Yangling, China

**Keywords:** inflammatory diet, gut microbiota, sensorineural hearing loss, NHANES, Mendelian randomization

## Abstract

**Aims:**

Inflammatory diets can trigger chronic inflammation and affect gut microbiota. However, the relationship between dietary preferences and sensorineural hearing loss (SNHL) remains unclear. This study aims to elucidate the relationship between different dietary preferences and sensorineural deafness.

**Methods:**

The Dietary Inflammation Index (DII) and SNHL were defined by data from the National Health and Nutrition Examination Survey (NHANES), and exploring their relationship. Using Mendelian randomization (MR) to analyze the relationship between 34 dietary preferences, 211 gut microbiota, and SNHL.

**Results:**

Smooth curve fitting indicated that the risk of SNHL increased with increasing DII score when the DII score was greater than 5.15. MR results suggest that a diet including both oily and non-oily fish can substantially reduce the risk of SNHL. Additionally, six specific gut microbiota were found to have significant causal relationship with SNHL.

**Conclusion:**

An inflammatory diet may increase the risk of developing SNHL. The observed relationship between fish consumption, gut microbiota, and SNHL suggests the existence of a gut-inner ear axis.

## 1 Introduction

Hearing plays a critical role in the animal kingdom, aiding predators in locating their prey and helping animals avoid natural predators. In human society, hearing significantly impacts language and cognition. Research indicates that hearing loss in children can lead to impaired oral expression, delayed language development, and reduced literacy skills (1). In older adults, age-related hearing loss is common and can contribute to functional decline and loss of independence. Studies suggest that long-term hearing loss may lead to atrophy in certain brain areas, particularly the temporal lobe, which is responsible for auditory perception, language, and memory functions. Additionally, hearing loss can result in social isolation, increasing the risk of dementia (2). Unfortunately, hearing loss is a highly prevalent sensory disorder globally. Over 5% of the world’s population, including 34 million children—require rehabilitation for disabling hearing loss. It is estimated that by 2050, nearly 2.5 billion people will have some degree of hearing loss. Of these, more than 700 million will have disabling hearing loss (3). These issues pose significant economic and health challenges to society and public health. The etiology of sensorineural hearing loss (SNHL) is complex, often associated with aging, genetic mutations, noise exposure, ototoxic drugs, and degenerative processes linked to chronic diseases. Therefore, developing effective interventions to reduce or delay the onset of hearing loss remains a crucial component of the solution.

The relationship between inflammation and age-related diseases has received much attention in recent years. Unlike the acute inflammatory response, which is typically associated with infection and tissue injury and involves the recruitment of leukocytes and plasma proteins to the affected tissues, tissue stress or dysfunction triggers an adaptive response known as para-inflammation (4). This response is largely dependent on tissue-resident macrophages. Para-inflammation may be the culprit associated with modern human diseases. Chronic inflammation, a hallmark of immune senescence, is a mild inflammatory condition that exacerbates with age (5). Evidence of chronic inflammation has been observed in various models of aging-related diseases, including type II diabetes, cardiovascular disease, and Alzheimer’s disease (6–8). Chronic inflammation is closely linked to macrophages. Recent studies have identified resident macrophages in the cochlea, particularly within the spiral ligament, spiral ganglion, and stria vascularis. These tissue-resident macrophages play a crucial role in the detection, phagocytosis, and clearance of cellular debris and pathogens, in addition to triggering inflammation and affecting tissue repair through the production of inflammatory cytokines and chemokines. Cochlear injury can activate these macrophages, initiating an immune response (9). Previous research has established a strong association between diet and systemic inflammation. The Mediterranean diet, in particular, is renowned for its anti-inflammatory effects (10). In contrast, the gut microbiota, regulated by dietary practices, plays a key role in the host’s energy homeostasis, immune activity, and interactions with other body organs (11–14). Studies have confirmed that changes in gut flora are associated with the progression of SNHL (15). Pathological stress-induced inflammatory intestinal microenvironment may lead to the disruption of the intestinal barrier, which in turn allows metabolites and pro-inflammatory factors of the intestinal microbiota to be transferred to other organs, including the inner ear, via the body circulation (16). These findings may suggest the presence of a gut-inner ear axis. Given this background, our study aims to explore the association between dietary inflammation and sensorineural deafness.

Diet influences the risk of developing chronic diseases through multiple mechanisms, such as oxidative stress modulation, energy balance regulation, and alterations in gut microbiota (17, 18). These effects are due to dietary patterns and the pro- or anti-inflammatory properties of individual dietary components. Adopting a healthy dietary pattern and consuming nutrient-rich food groups have been shown to reduce inflammatory markers (19). The Dietary Inflammation Index (DII) offers a novel tool for exploring the inflammatory contributions of various dietary components. Although previous studies have established the role of the DII in the pathophysiology of neurodegenerative diseases, the relationship between inflammation-related sensorineural hearing loss and the DII remains unclear. To address this gap, we explored the relationship between DII and SNHL using National Health and Nutrition Examination Survey (NHANES) data. Additionally, we conducted two multi-omics Mendelian randomization (MR) studies to investigate the effects of different dietary preferences and gut microbiota on SNHL.

## 2 Methods

Our study comprises two main parts. First, we utilized dietary and hearing data from the NHANES database to define the DII and assess the presence of SNHL in subjects. Using multivariate logistic regression, smoothed curve analysis, and subgroup analyses, we investigated the relationship between DII and SNHL. Second, the instrumental variables (IVs) were extracted from genome-wide association studies (GWAS) related to dietary preferences. We used a two-sample MR method to assess the effect of various dietary preferences on susceptibility to SNHL. Similarly, we extracted IVs for 211 species of gut microbiota to explore the causal relationship between human gut microbiota and SNHL, thereby corroborating the existence of the gut-inner ear axis.

### 2.1 Cross-sectional study

#### 2.1.1 Description of data sources

**Figure 1:**
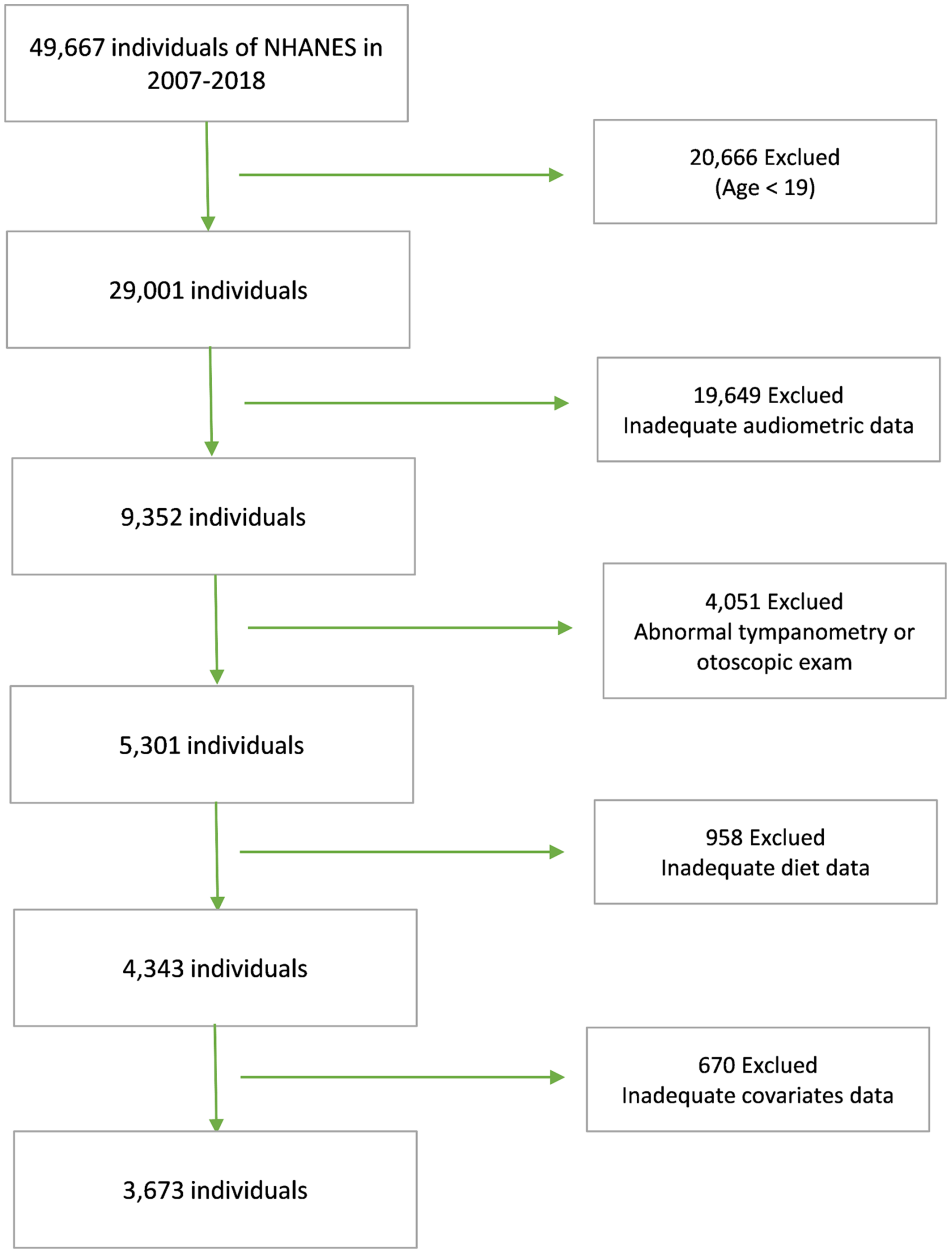
Flow chart of study participants.

NHANES is a national program designed to assess the health and nutritional status of Americans. Conducted biennially, NHANES boasts a sample size of approximately 5,000 individuals and adheres strictly to research ethics principles. To explore the relationship between DII and SNHL, data from five NHANES cycles, 2007 through 2012 and 2015 through 2018, were used. These cycles were chosen due to their comprehensive coverage of the variables necessary for both dietary and hearing data, with all data meticulously processed using standardized protocols. Our analyses adhered rigorously to predetermined exclusion criteria, which encompassed individuals under the age of 19, those exhibiting abnormal findings in the tympanic chamber or otoscopic examinations, as well as those lacking complete dietary, hearing, or covariate information. Initially, our participant pool comprised 49,667 individuals. However, after applying these stringent exclusion criteria, our study ultimately encompassed 3,673 participants (Figure 1).

#### 2.1.2 Diet inflammatory index

The DII is a validated tool derived from literature, comprising 45 food parameters recognized for their anti-inflammatory or pro-inflammatory properties. It serves to standardize the classification of individual dietary components. This tool has been employed to forecast levels of inflammatory markers across various datasets and populations. Consistent with prior research, we extracted 26 relevant food parameters from the NHANES database and computed the DII for each dietary component using a standardized method. This calculation involved determining the standard deviation of (daily intake - global mean daily intake) divided by the global mean daily intake, multiplied by the overall inflammatory effect score of the respective dietary component (20). The summation of these individual DIIs yields the subject’s overall DII. Detailed information on the dietary components utilized in calculating the DII is provided in Supplementary Table S1.

#### 2.1.3 SNHL defined

SNHL can be defined when the mean pure tone hearing threshold exceeds 20 dB and potential mixed or conductive hearing loss is excluded (21). In accordance with established criteria and prior literature, the following parameters were employed to diagnose SNHL in this study: mean pure tone hearing thresholds exceeding 20 dB, normal otoscopic findings, peak conductance of at least 0.3 mL, tympanograms indicative of type A, and denial of a history of colds within 24 h (22). The NHANES audiometric assessment program encompasses audiometric questionnaires, otoscopy, pure-tone air-conduction audiometry, and tympanometry. Trained examiners administer all components of the audiometric examination to participants within a dedicated soundproof room at the Mobile Examination Center (MEC). During audiometry, careful evaluation of air-conduction thresholds is conducted for each ear across seven frequencies, with intensity levels ranging from −10 to 120 dB. Additionally, thresholds for each ear are repeated at 1,000 Hz, spanning an intensity range of −10 to 120 dB. If the disparity between the results of the two tests exceeds 10 dB, the results are deemed unacceptable; conversely, if the variance falls within 10 dB, the outcomes of the initial 1,000 Hz test are utilized.

#### 2.1.4 Covariates

The covariates used in this study were derived from demographic and health-related data in NHANES and included age, race, gender, household income, educational attainment, poverty rate, diabetes status, and body mass index (BMI). In addition, serum cotinine has a longer half-life in the blood and is viewed as a marker of active smoking (23, 24). Detailed data for all variables in the study are available at www.cdc.gov/nchs/nhanes/.

### 2.2 Mendelian randomization

#### 2.2.1 Data availability

The MR analysis utilized publicly available data. GWAS data pertaining to dietary patterns were sourced from the GWAS Catalog, specifically entries GCST90096892-GCST90096929 (25). These datasets comprised information from 445,779 participants enrolled in the UK Biobank, identifying 283 genetic markers associated with dietary intake. To isolate direct genetic effects on food exposure, a total of 38 GWAS for dietary preferences were identified after adjusting for effects mediated through other traits. Detailed information is provided in Supplementary Table S1. GWAS datasets concerning the composition of the human gut microbiota were from the international consortium MiBioGen (26). This extensive GWAS study involved 24 ethnic cohorts and included genotyping data from 18,340 participants, exploring associations between human genetic variation and the gut microbiota. The GWAS analyses included a total of 211 taxa covering 131 genera, 35 families, 20 orders, 16 classes, and 9 phyla.

Furthermore, GWAS data related to SNHL were accessible from the FinnGen R9 consortium, comprising 32,487 cases and 331,736 controls. Within this dataset, sensory deafness was defined as hearing loss originating from the inner ear or sensory organs or the vestibular nerve.

#### 2.2.2 Selection of IVs

We screened for independent single nucleotide polymorphisms (SNPs) that constitute instrumental variables (IVs) associated with 38 dietary preferences and 211 human gut microbiota. To select independent genetic variants, genome-wide significant SNPs were grouped by linkage disequilibrium (LD) (*r*^2^ < 0.001 for SNPs within a 1 Mb genomic region). Since the gut microbiota GWAS was unable to screen a sufficient amount of IVs after the stringent screening criteria described above, we followed the same criteria as previous studies and relaxed the correlation screening criteria to *p* < 1 × 10^−5^ (27, 28). Finally, a two-sample MR was performed using IVs separately and SNHL GWAS data.

#### 2.2.3 Statistical analysis

MR uses genetic variation as a tool to test the causal relationship between an exposure (dietary preference and gut microbiota) and an outcome (SNHL) that requires three core assumptions to be met. MR estimates for each risk factor were determined using inverse variance weighted (MR-IVW) analysis as the primary means of MR analysis, which uses random effects meta-analysis to combine Wald ratio estimates of causal effects obtained from each SNPs tested. We conducted a series of sensitivity analyses, including MR-Egger, weighted median, and heterogeneity tests to test the underlying assumptions of MR.

## 3 Results

### 3.1 Cross-sectional study

#### 3.1.1 Baseline characteristics

In our study, data were collected from a total of 3,673 participants, with a mean age of 45.83 ± 16.17 years. Among these participants, 47.07% were males and 52.93% were females. As depicted in Table 1, we categorized the survey-weighted participant characteristics into two groups based on disease status: SNHL and normal. Of the total participants, 1,742 (47.43%) exhibited SNHL. A comparative analysis revealed that individuals with SNHL were more likely to belong to older age groups, male gender, non-Hispanic white ethnicity, have lower levels of educational attainment, be at risk for diabetes, have a history of tobacco use, and possess a higher body mass index, in comparison to those without SNHL.

**Table 1:** Clinical characteristics of all 3,673 subjects among subjects with SNHL and without SNHL.

Characteristics	Control	SNHL	*p*-value
	*n* = 1931	*n* = 1742	
Age, years	35.97 ± 11.04	55.15 ± 13.44	<0.0001
Gender (%)			<0.0001
Male	42.18	52.99	
Female	57.82	47.01	
Race (%)			<0.0001
Mexican American	9.51	6.35	
Other Hispanic	7.03	5.43	
Non-Hispanic White	64.64	76.06	
Non-Hispanic Black	11.27	6.94	
Other race	7.56	5.23	
Education level (%)			<0.0001
<9th grade	2.55	4.51	
9–11th grade	7.03	9.63	
High school grade/GED or equivalent	15.97	23.70	
Some college or AA degree	32.87	30.52	
College graduate or above	41.58	31.64	
Diabetes mellitus (%)	3.37	14.08	<0.0001
PIR, mean	3.03 ± 1.66	3.19 ± 1.61	0.0038
Cotinine, ng/mL	41.75 ± 103.32	56.00 ± 132.64	0.0003
BMI, kg/m^2^	28.42 ± 6.94	29.84 ± 6.60	<0.0001

Mean ± SD for continuous variables: the *p*-value was calculated by weighted linear regression model. PIR, the ratio of family income to poverty; BMI, body mass index; SNHL, sensorineural hearing loss.

#### 3.1.2 The association between DII and SNHL

Our findings revealed that there was no significant correlation between DII and SNHL in either the unadjusted logistic regression model or the multivariate logistic regression model after accounting for various covariates (Table 2). However, when attempting to model the nonlinear relationship between SNHL and DII by fitting a smoothed curve, we observed a similar U-shaped correlation between DII score and SNHL, even after adjusting for different covariates. In particular, through a threshold effect analysis, we noted that the risk of hearing loss was associated with higher DII scores when the DII score exceeded 5.15 (Table 3).

**Table 2:** The associations between DII and SNHL.

Exposure	Model I OR (95% CI) *P*	Model II OR (95% CI) *P*	Model III OR (95% CI) *P*
DII	0.99 (0.97, 1.00) 0.0164	1.01 (0.99, 1.02) 0.2270	0.99 (0.98, 1.01) 0.3526

Model I: None covariates were adjusted. Model II: Gender, age and race were adjusted. Model III: Gender, age, race, education level, PIR, Cotinine, BMI and Diabetes were adjusted. PIR, the ratio of family income to poverty; BMI, body mass index; DII, Dietary Inflammation Index; SNHL, sensorineural hearing loss.

**Table 3:** Threshold effect analysis of DII and SNHL.

	Adjusted HR (95% CI), *p*-value
Fitting by the standard linear model	0.99 (0.98, 1.01) 0.3526
Fitting by the two-piecewise linear model	
Inflection point	5.15
DII < 5.15	0.98 (0.96, 1.00) 0.0305
DII ≥ 5.15	1.14 (1.04, 1.26) 0.0068
*P* for Log-likelihood ratio	0.004

Gender, age, race, education level, BMI, PIR, Cotinine and Diabetes were adjusted. PIR, the ratio of family income to poverty; BMI, body mass index; DII, Dietary Inflammation Index; SNHL, sensorineural hearing loss.

#### 3.1.3 Subgroup analyses

No interaction between the unfavorable correlation between DII and SNHL with age, gender and diabetes was found in this study (Table 4).

**Table 4:** Subgroup analysis for the association between DII and SNHL.

Subgroup	OR (95%CI)	*P* for interaction
Gender		0.8064
Male	0.99 (0.98, 1.01) 0.3830	
Female	1.00 (0.98, 1.02) 0.7329	
Age		0.6260
<65	0.97 (0.97, 0.99) <0.0001	
≥65	0.99 (0.92, 1.07) 0.8666	
Diabetes status		0.9231
Yes	0.98 (0.94, 1.03) 0.5155	
No	0.98 (0.97, 0.99) 0.0043	

DII, Dietary Inflammation Index; SNHL, sensorineural hearing loss.

### 3.2 Mendelian randomization

We used three methods to assess the causal relationship between dietary preferences and SNHL, with IVW as the primary means of MR analysis. Thirty four dietary preferences and 211 gut microbiota were screened for SNPs used for genetic prediction. The F-statistics of these genetic tools were all greater than the threshold of 10, indicating stronger tools.

After a comprehensive MR analysis, we found that a fish diet significantly reduced the risk of sensorineural deafness. Among them, consumption of non-oily fish (OR = 0.068, 95%CI: 0.018–0.259, *p* = 8.541 × 10^−5^) as well as oily fish (OR = 0.558, 95%CI: 0.386–0.807, *p* = 1.921 × 10^−3^) showed a protective effect on hearing. The *p*-value of MR-Egger intercept for both positive MR analyses was greater than 0.05, indicating that no pleiotropy was detected. The p-value of the Cochran Q test for the non-oily fish diet was 0.323, but the p-value of the Cochran Q test for the oily fish diet was less than 0.05, which may indicate heterogeneity. However, the heterogeneity was acceptable because the IVW method of random effects model was used as an assessment tool in this study (29).

In addition to this, after performing 211 MR analyses, we screened out six gut microbiota that were significantly associated with SNHL, with varying effect values. The genus *RikenellaceaeRC9gutgroup* (OR = 1.056, *p* = 0.025), genus *Bifidobacterium* (OR = 1.131, *p* = 0.004), and family *Porphyromonadaceae* (OR = 1.159, *p* = 0.018) showed positive correlation for SNHL; The phylum *Verrucomicrobia* (OR = 0.912, *p* = 0.015), genus *Flavonifractor* (OR = 0.878, *p* = 0.021), and family *Streptococcaceae* (OR = 0.919, *p* = 0.047) showed negative correlation for SNHL. Detailed information on all positive endpoints is displayed in Table 5. We performed sensitivity analyses on all results, and scatter plots, funnel plots, and “leave one-out analysis” plots can be viewed in Supplementary Figure S1.

**Table 5:** Significant estimates for MR analysis.

Exposure	Outcome	IVW-derived *p*-value	OR (95% CI)	Cochran’s Q-derived *p*-value	MR-Egger intercept derived *p*-value
Non-oily fish consumption	SNHL	8.541 × 10^−5^	0.068 (0.018–0.259)	0.323	0.796
Oily fish consumption	SNHL	1.921 × 10^−3^	0.558 (0.386–0.807)	5.358 × 10^−4^	0.374
*Verrucomicrobia*	SNHL	0.015	0.912 (0.848–0.982)	0.879	0.624
*RikenellaceaeRC9gutgroup*	SNHL	0.025	1.056 (1.007–1.108)	0.541	0.399
*Flavonifractor*	SNHL	0.021	0.878 (0.786–0.980)	0.323	0.515
*Bifidobacterium*	SNHL	0.004	1.131 (1.039–1.230)	0.056	0.586
*Streptococcaceae*	SNHL	0.047	0.919 (0.845–0.999)	0.196	0.510
*Porphyromonadaceae*	SNHL	0.018	1.159 (1.026–1.310)	0.397	0.614

SNHL, sensorineural hearing loss; IVW, inverse variance weighted.

## 4 Discussion

In this study, we analyzed data from large observational studies and GWAS to evaluate the influence of inflammatory dietary preferences on SNHL. We then conducted a multi-omics MR analysis to investigate the causal impact of gut microbiota on SNHL. Our analysis revealed that an inflammatory diet indeed raises the risk of SNHL. Subsequently, our MR analysis found that a diet rich in fish significantly reduced the risk of SNHL. In addition, we identified 6 specific gut microbiota that were significantly associated with SNHL. Overall, our study confirms the strong relationship between diet and SNHL and provides compelling evidence supporting the existence of a gut-inner ear axis.

Inflammation underlies many pathophysiological processes, typically triggered by infection and injury. Recently, the concept of quasi-inflammation has gained attention. This adaptive response occurs when tissues react to various stimuli—such as pathogens, cellular debris, nutrients, and intestinal microbiota—in a manner that lies between homeostasis and classical inflammation (4). Quasi-inflammation may contribute to numerous chronic diseases in modern humans, partly due to shifts in the body’s homeostatic set-points (e.g., insulin sensitivity) (4). Unlike acute inflammation, which requires significant tissue damage or infection, quasi-inflammation is prompted by tissue dysfunction and aims to restore function and homeostasis. Aging and gut microbiota dysbiosis are linked to para-inflammation, with age-related immune deficiencies leading to unresolved inflammatory processes. Both aging and high-fat diets increase intestinal permeability, allowing leakage that can provoke systemic inflammation (16, 30–32). It has been proposed that inflammation from gut dysbiosis accelerates age-related cochlear degeneration (33). Diet plays a crucial role in regulating individual inflammation levels. Several randomized controlled trials have shown that the Mediterranean diet is associated with lower concentrations of the inflammatory marker C-reactive protein (CRP) (34), whereas unhealthy foods that are high in energy, fat and sugar, and low in dietary fiber may contribute to local and systemic inflammation (35). And it has long been shown that chronic inflammation is associated with most human pathologies, such as cancer and autoimmune and neurodegenerative diseases (36). We also chose the DII because the index is universal, involving the 6 most commonly studied markers of inflammation, and can be used as a summary measure of diet-related inflammation in any population (37). According to our results, the risk of hearing impairment increases when the DII exceeds a certain threshold, which is consistent with the previous belief that an inflammatory diet leads to chronic disease. Based on previous studies and our current analysis of the NHANES data, we believe that reducing inflammatory diets and optimizing dietary strategies are essential for the prevention and control of chronic diseases, including age-related hearing loss. Moreover, the protective effect of a fish-rich diet against sensorineural deafness has been consistently supported by observational studies (38, 39). Good blood flow within the cochlea is crucial for ensuring the delivery of adequate oxygen and glucose while efficiently removing metabolic byproducts. An impaired blood supply to the cochlea can disrupt the maintenance of intracochlear potential, endolymphatic fluid balance, and the integrity of the blood-labyrinth barrier (BLB). These disruptions can lead to hypoxic–ischemic damage to hair cells and subsequent hearing loss (40). Additionally, cochlear hypoperfusion-induced mitochondrial DNA damage and chronic inflammation have been associated with age-related hearing loss (41, 42). The intake of fish and long-chain omega-3 polyunsaturated fatty acids may help mitigate these impairments and slow the progression of age-related hearing loss. This protective effect occurs through various mechanisms, including improving vascular reactivity and endothelial function, preventing thrombosis and inflammation, modulating membrane ion channels and electrophysiological responses to ischemic stress, influencing gene expression, and reducing pro-inflammatory or pre-thrombotic eicosanoids derived from arachidonic acid (43). Our study aligns with the findings of a previous prospective study that followed 1,038,093 subjects over several years. This study demonstrated that consuming two or more servings of fish per week reduced the risk of hearing loss, and that higher intake of long-chain omega-3 polyunsaturated fatty acids was also negatively associated with the risk of hearing loss (39). However, it is noteworthy that the prior study’s conclusions were based on US females, and our MR analyses did not perform gender subgroup analyses. Additionally, the previous study confirmed that consumption of any type of fish tended to reduce the risk of hearing loss, suggesting that the benefits may be largely attributable to the omega-3 fatty acids found in fish. Interestingly, our study found that the consumption of non-fatty fish provided more hearing protection than fatty fish, indicating that other potential protective pathways might exist. These pathways may involve complex interactions between various nutrients in fish that act synergistically with omega-3 fatty acids to protect against hearing loss. This finding complements previous observational studies and suggests that a diet rich in fish can be an effective strategy for preserving hearing health.

The balance of the gastrointestinal tract is closely linked to the health of many distal organs. For example, the gut-brain axis and gut-lung axis are well-established, and gut microbiota play a crucial role in these interactions (44–47). Bacteria within the gut can exhibit both pro-inflammatory and anti-inflammatory properties, and the gut microbiota responds dynamically to different diets. When the balance between microorganisms is disturbed, it can lead to ecological imbalances and various inflammatory responses, increasing the host’s susceptibility to diseases (48). The gut microbiota communicates with the host through multiple pathways, including microbial metabolites, tryptophan metabolism and the immune system. These interactions can trigger the secretion of chemokines, neurotransmitters, cytokines, neuropeptides, endocrine messengers and microbial byproducts. These molecules can enter the vascular and lymphatic systems, influencing neural signals transmitted by vagal and spinal afferents. Chronic systemic inflammation can result from dysbiosis of the intestinal flora, leading to disrupted gut ecology, increased intestinal barrier permeability, and the infiltration of pathogens and microbial solutes into the bloodstream (30). This chronic inflammation can compromise the integrity of the blood–brain barrier (BBB), allowing pathogens and pro-inflammatory cytokines to infiltrate the brain, which can lead to neuroinflammation and neurodegeneration (49–51).

**Figure 2:**
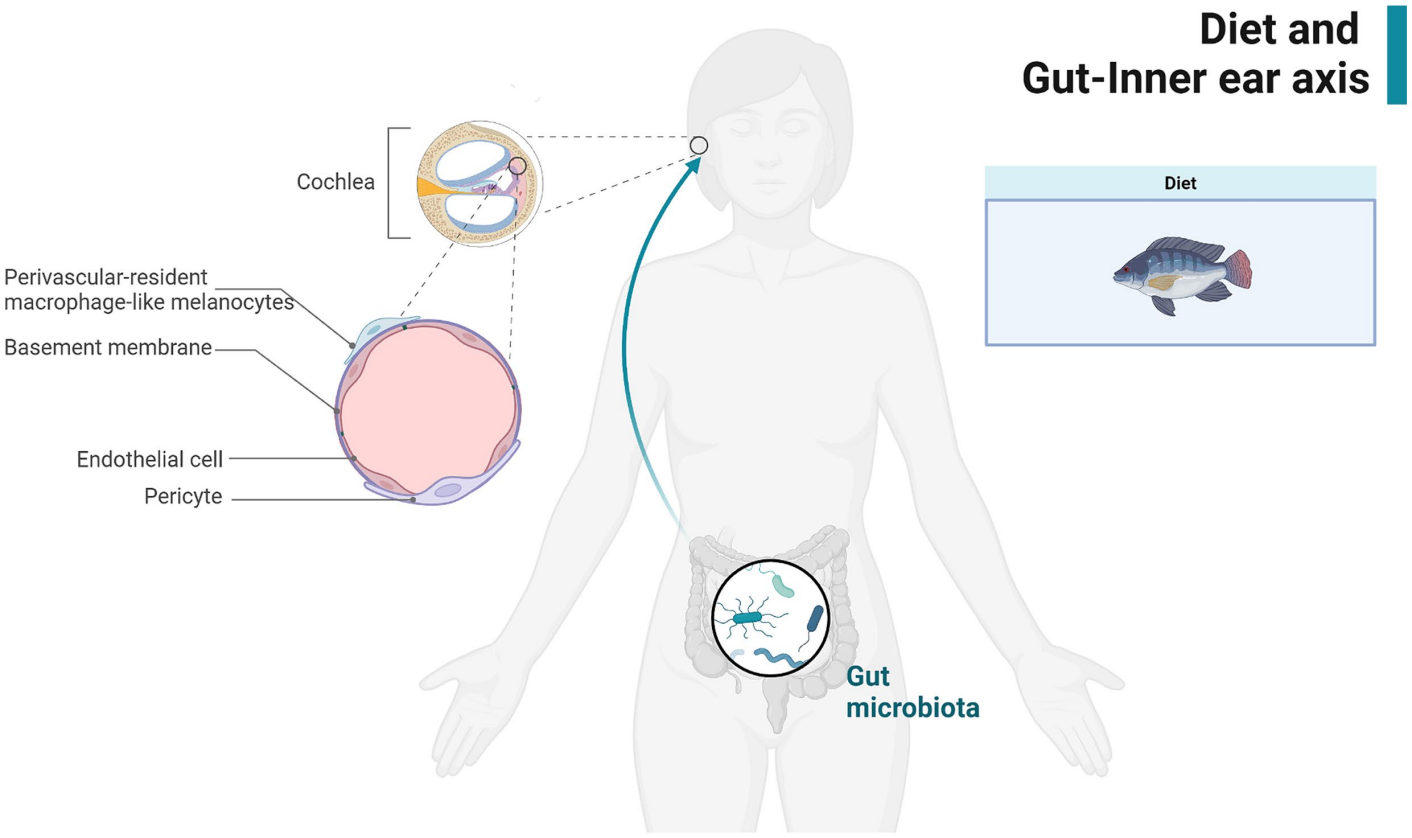
Demonstration of the gut-inner ear axis. Created with BioRender.com.

The BLB in the stria vascularis of the inner ear consists of pericytes (PCs), vascular endothelial cells (ECs), and Perivascular-resident macrophage-like melanocytes, is analogous to the BBB, making it susceptible to the influence of metabolites from the gut microbiota and pro-inflammatory molecules. These substances can penetrate the BLB, potentially compromising cochlear integrity and leading to inflammation and injury (16, 52). Dysfunction of the BLB due to inflammation can reduce its ability to restrict the entry of inflammatory or infectious agents and immune cells into the cochlea, thereby exacerbating cochlear damage (53, 54). Nearly all major causes of acquired hearing loss share similar pathogenic mechanisms, suggesting a potential link between dietary preferences, gut microbes, and inner ear health (Figure 2). Three of the six gut microbiota found in our study to be significantly associated with SNHL increase susceptibility to SNHL. The association between the *RikenellaceaeRC9gutgroup* and inflammation and obesity has long been established (55, 56). The *Porphyromonas* family is also thought to be associated with increased intestinal permeability and a shift in features associated with inflammatory diseases (57). However, it is puzzling that *Bifidobacteria* associated with anti-inflammation proved in our study to also increase susceptibility to SNHL. Inflammatory diets have been shown to decrease *Flavonifractor* abundance thereby affecting glucose homeostasis and increasing the systemic inflammatory state (58). This is the same conclusion as our current study. This relationship may be explained by chronic inflammation and the emerging concept of the gut-inner ear axis. For instance, an observational study found that the prevalence of high-frequency hearing loss was significantly higher in obese adolescents compared to their normal-weight peers (59). Additionally, serum triglycerides and blood glucose levels have been associated with sensorineural hearing loss, potentially related to cochlear blood supply (60). Our study adds to the growing body of evidence supporting the gut-inner ear axis by elucidating possible mechanisms linking gut microbiota and dietary factors to cochlear health. This connection underscores the importance of maintaining gut health to prevent or mitigate hearing loss.

Our study has significant strengths. First, using a large sample from NHANES, we defined the DII index using dietary data and inferred a potential relationship between the DII index and sensorineural deafness. In addition, we further inferred the effect of dietary preference and gut microbiota on susceptibility to SNHL at the genetic level using MR analysis, excluding confounders, and based on this we confirmed and delved into the existence of the gut-inner ear axis. This is a more complete study of the correlation between diet and SNHL at present. Our study also has limitations. First, data obtained from dietary questionnaires in observational studies may be biased. Dietary questionnaires may have errors in reporting and recall, subjects with lower DII scores may have healthier dietary and lifestyle habits, and despite our adjustment for covariates, there are possible confounders that could not be controlled for. Second, the *p*-values of both multi-omics MRs for dietary preference and gut flora were not corrected for, which may present the possibility of false positives. However, previous histologic MR studies have concluded that even if the corrected p-value is greater than 0.05, it should still be considered suggestive of a potential association (61, 62). The GWAS data in this study were all from European populations, so the conclusions from the MR analyses apply only to Europeans, and similarly, the NHANES data were from US populations, and the conclusions about this section apply only to Americans. The generalizability of the conclusions would need to be verified in the future using other populations (e.g., Asian or African). In the end, the present study only explored the relationship between inflammatory diet and gut flora and SNHL from observational studies and genetic perspectives, and more basic studies are needed in the future to confirm and elucidate the specific mechanisms and pathways of these causative relationships, so as to establish a detailed mechanism of the gut-inner ear axis.

## Data Availability

The original contributions presented in the study are included in the article/Supplementary material, further inquiries can be directed to the corresponding authors.
