# Supplementary material for: Inflammatory diet, gut microbiota and sensorineural hearing loss: a cross-sectional and Mendelian randomization study
Source: Front Nutr. 2024 Aug 16;11:1458484. doi: 10.3389/fnut.2024.1458484 (PMC11363541; doi:10.3389/fnut.2024.1458484)

Supplementary Figure

Inflammatory diet, Gut microbiota and Sensorineural hearing loss: a Cross-sectional and Mendelian randomization study

Yixuan Wang, Jiayi Nie, Kaige Yan, Jing Wang, Xin Wang*, Yuxiang Zhao*

Results of dietary preferences on SNHL

Figure S1. Scatter plots, Leave-one-out plots and Funnel plots for IVW estimates of significant results from non-oily fish on SNHL.

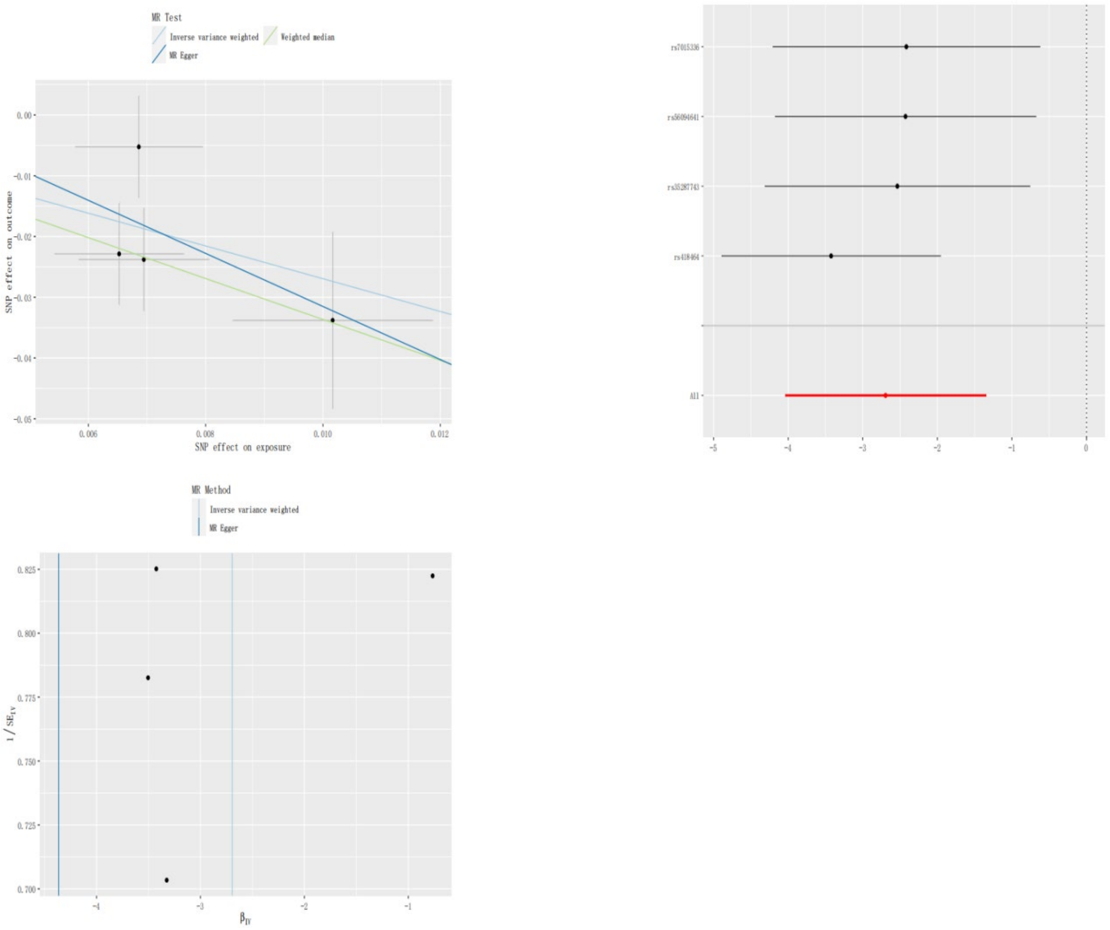

Figure S2. Scatter plots, Leave-one-out plots and Funnel plots for IVW estimates of significant results from oily fish on RA.

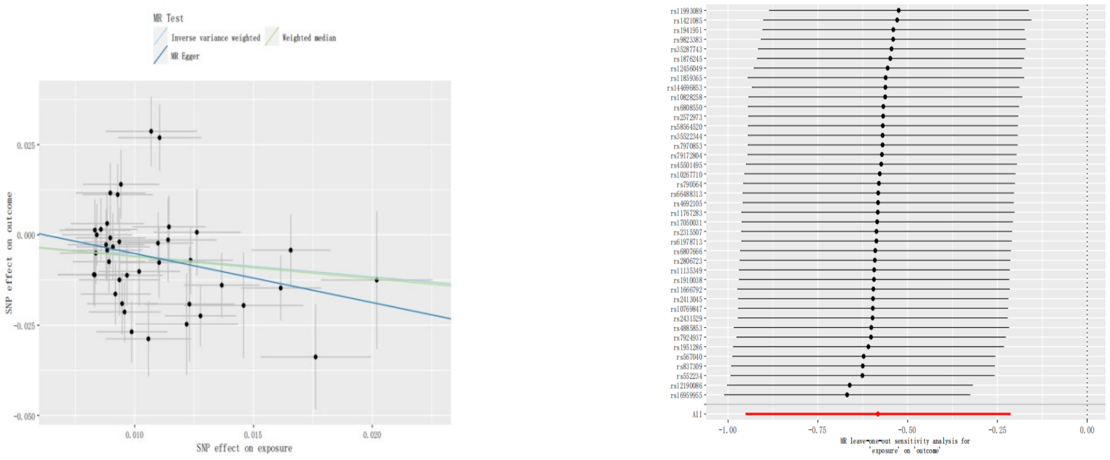

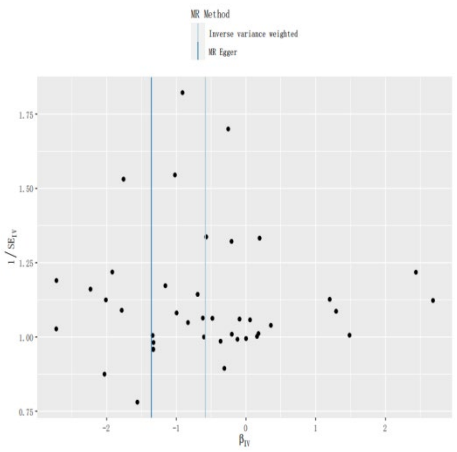

Figure S3. Scatter plots, Leave-one-out plots and Funnel plots for IVW estimates of significant results from Verrucomicrobia on SNHL.

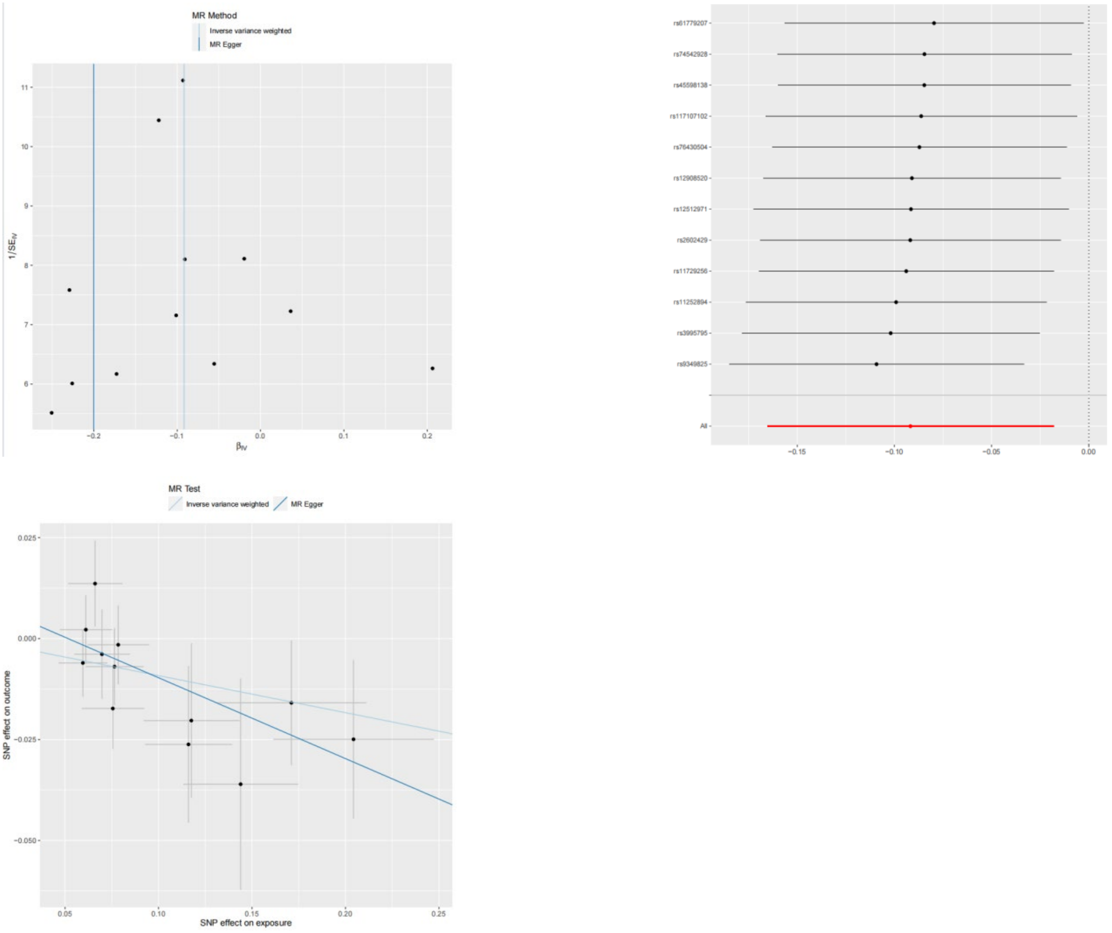

Figure S4. Scatter plots, Leave-one-out plots and Funnel plots for IVW estimates of significant results from RikenellaceaeRC9gutgroup on SNHL.

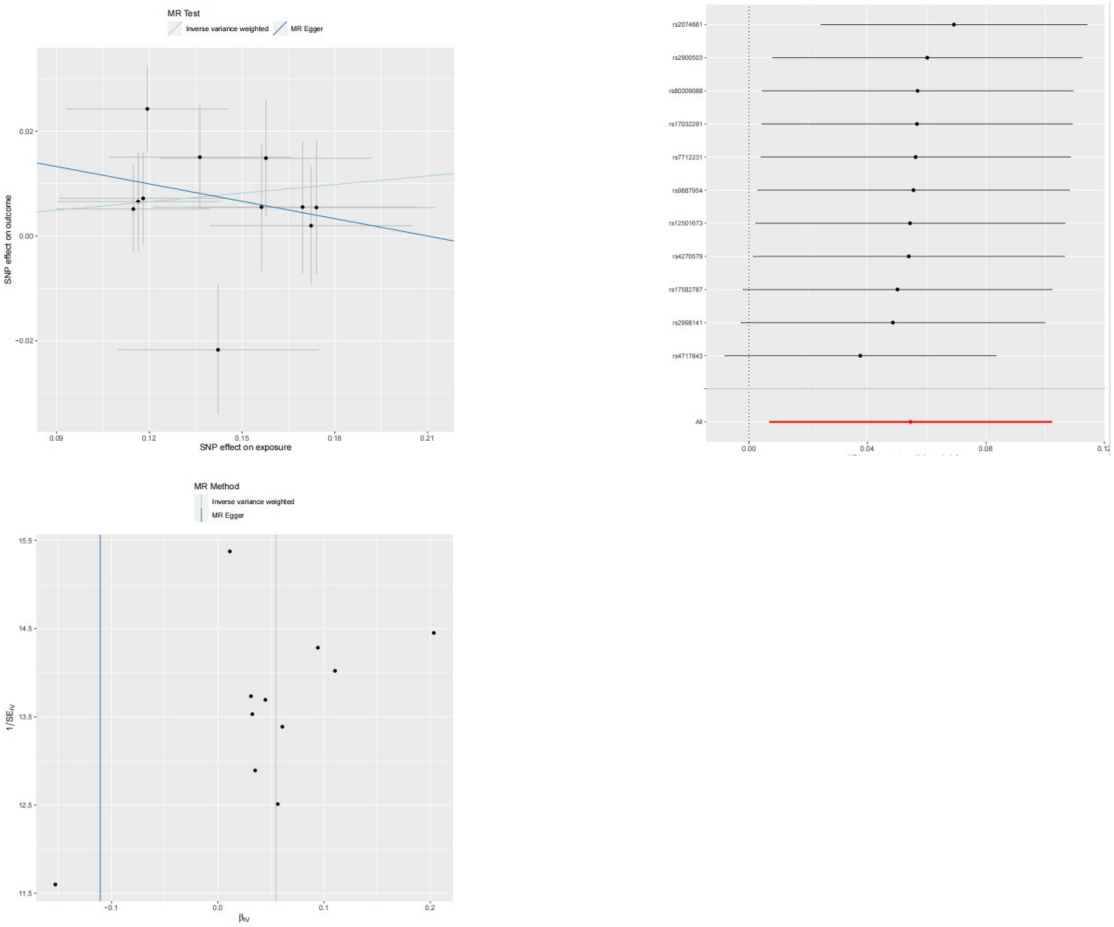

Figure S5. Scatter plots, Leave-one-out plots and Funnel plots for IVW estimates of significant results from Flavonifractor on SNHL.

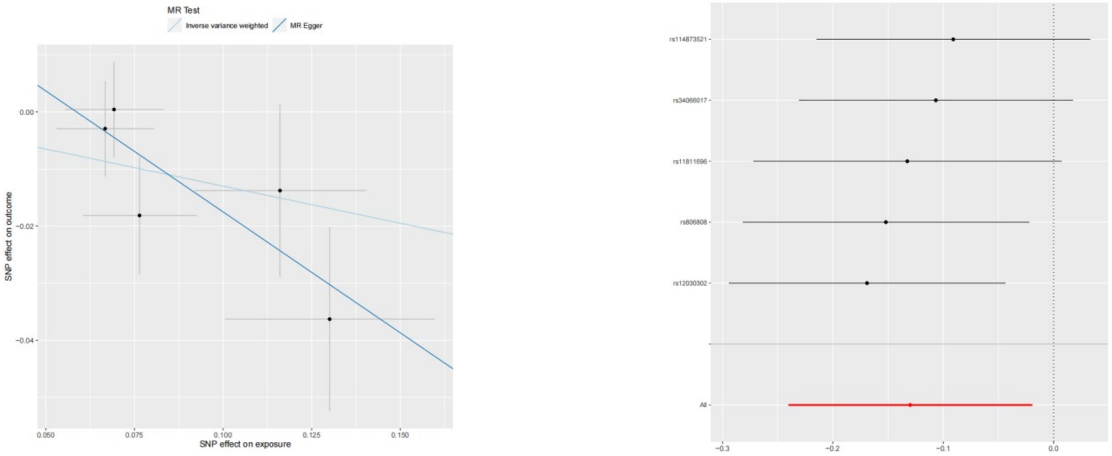

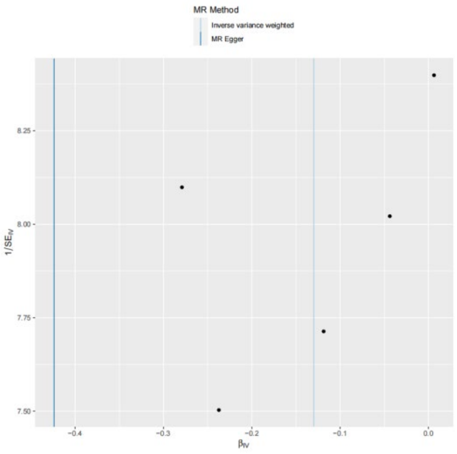

Figure S6. Scatter plots, Leave-one-out plots and Funnel plots for IVW estimates of significant results from Bifidobacterium on SNHL.

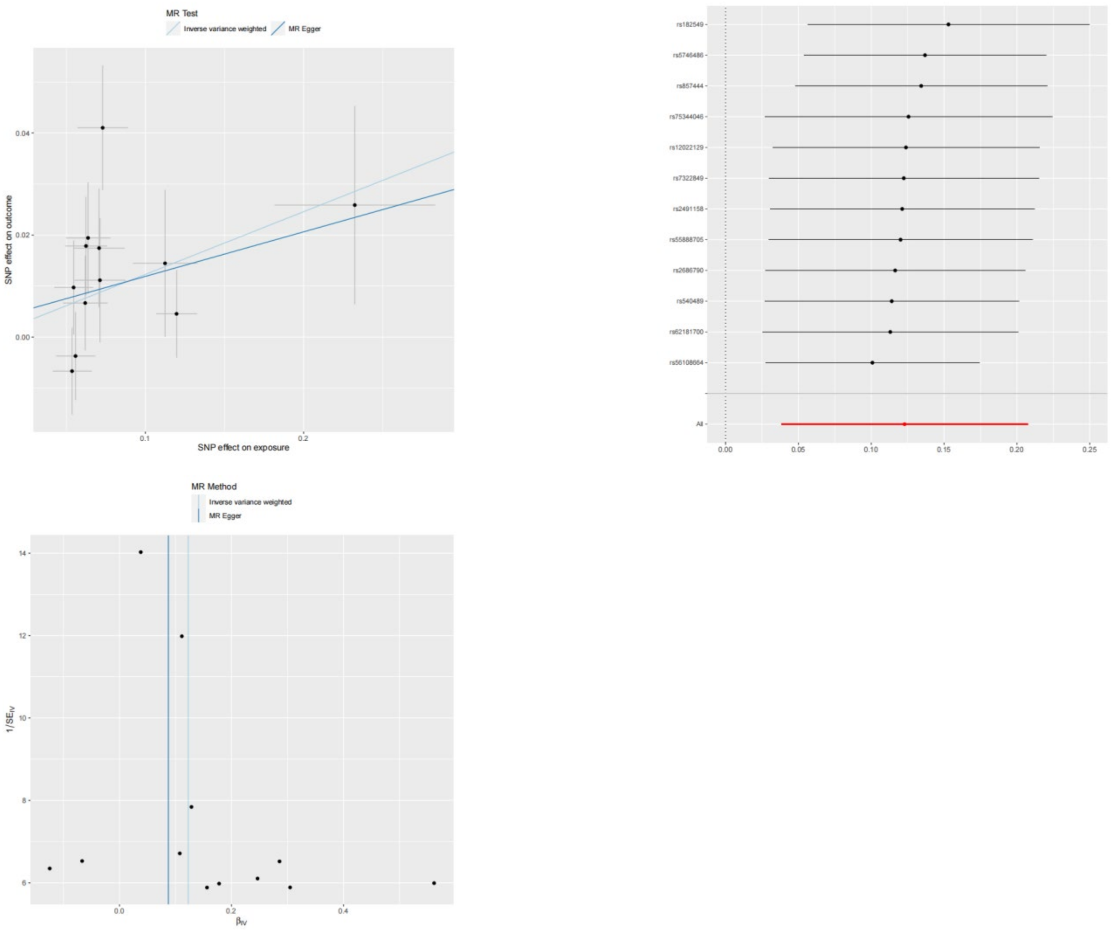

Figure S7. Scatter plots, Leave-one-out plots and Funnel plots for IVW estimates of significant results from Streptococcaceae on SNHL.

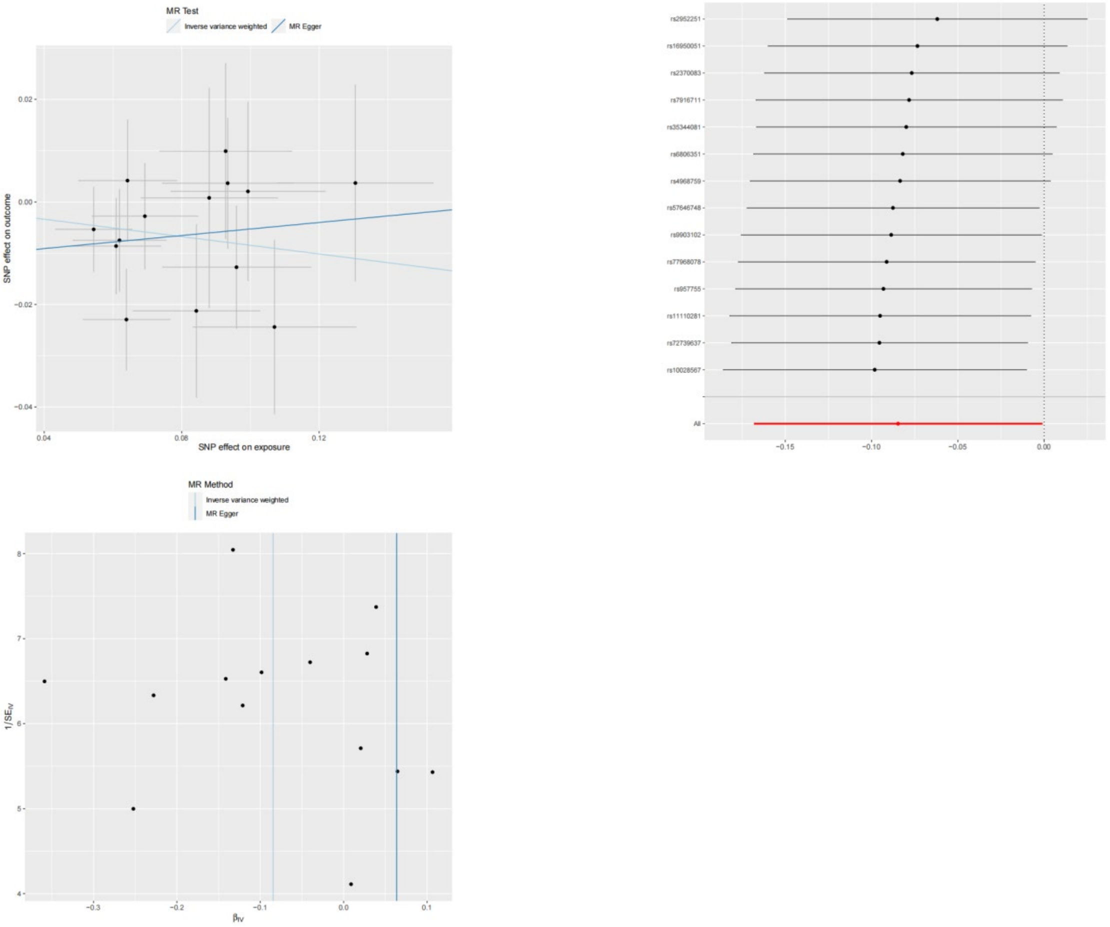

Figure S8. Scatter plots, Leave-one-out plots and Funnel plots for IVW estimates of significant results from Porphyromonadaceae on SNHL.

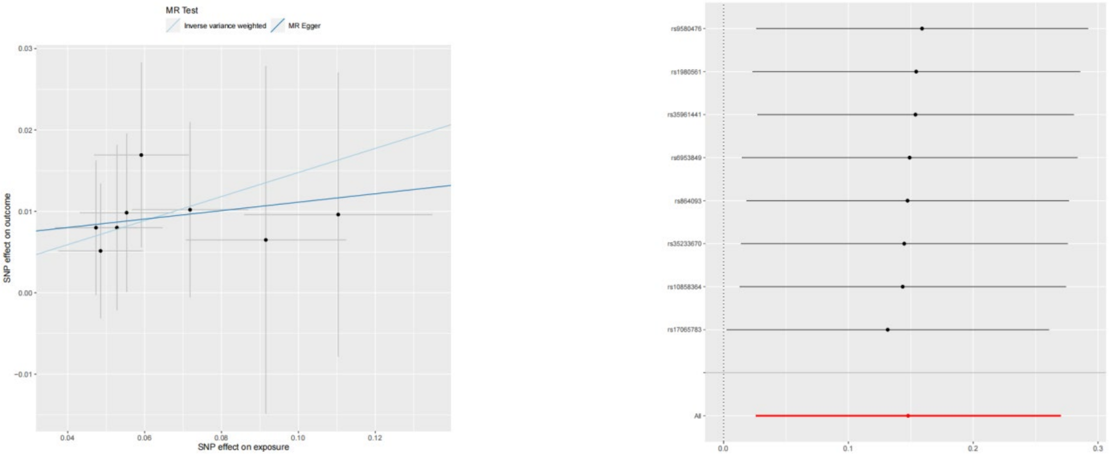

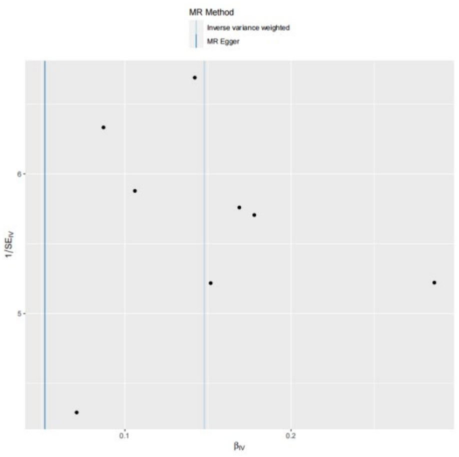

Figure S9. Subgroup analysis forest maps for the association between DII and SNHL.

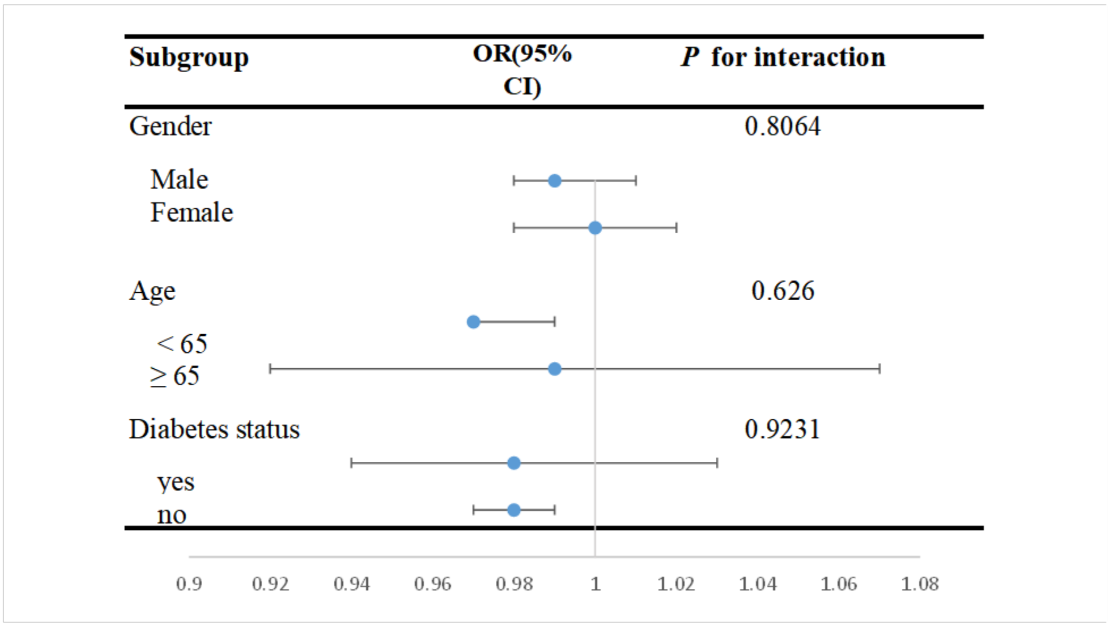

Figure S10. Smooth curve fitting for the association between DII and SNHL.

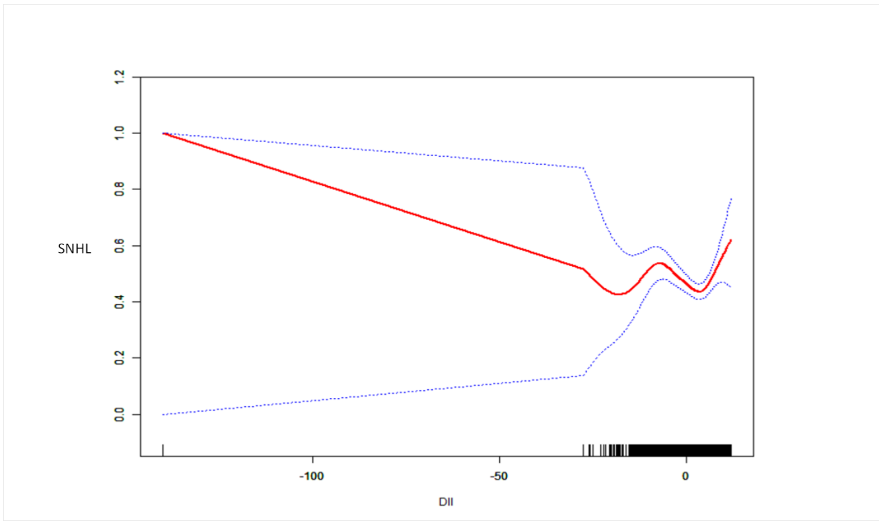

Supplement: Supplementary file 2 [file Image_1.pdf]
